# Open Reduction and Internal Fixation Is a Feasible Alternative to Femoral Revision Arthroplasty in Geriatric Patients with Vancouver B2/3 Type Periprosthetic Fractures: A Study Analyzing In-Hospital Outcomes

**DOI:** 10.3390/jcm13216475

**Published:** 2024-10-29

**Authors:** Christopher Lampert, Christoph Linhart, Boris Michael Holzapfel, Wolfgang Böcker, Carl Neuerburg, Yunjie Zhang

**Affiliations:** Department of Orthopedics and Trauma Surgery, Musculoskeletal University Center Munich (MUM), University Hospital, Ludwig Maximilian University of Munich, 81377 Munich, Germany; christopher.lampert@med.uni-muenchen.de (C.L.); christoph.linhart@med.uni-muenchen.de (C.L.); boris.holzapfel@med.uni-muenchen.de (B.M.H.); direktion-unfall@med.uni-muenchen.de (W.B.); carl.neuerburg@med.uni-muenchen.de (C.N.)

**Keywords:** periprosthetic fracture, osteosynthesis, Vancouver B, revision arthroplasty

## Abstract

**Purpose**: The surgical management of periprosthetic femoral fractures is particularly challenging in geriatric patients due to physiological limitations. The choice between open reduction and internal fixation (ORIF) and hip revision arthroplasty for treating Vancouver B2 and B3 fractures remains controversial. This study aims to contribute further evidence by analyzing the in-hospital outcomes in geriatric patients with Vancouver B2/3 fractures. **Methods**: This retrospective study analyzed 133 patients treated for Vancouver B2/3 fractures at a level I trauma center from 2017 to 2023. Data were collected on preclinical characteristics, comorbidities, Vancouver classification, surgery-related parameters, and postoperative outcomes for an age- and gender-matched analysis. A subgroup analysis was also conducted on patients classified as American Society of Anesthesiologists (ASA) class 3 and 4. **Results**: Among the 133 patients, 85 suffered Vancouver B2 fractures and 48 Vancouver B3 fractures. Age-and-gender-matched analysis revealed that ORIF was more commonly performed in patients with higher ASA grades. A subgroup analysis of ASA 3 and 4 patients and an age-and-gender-matched analysis showed that ORIF resulted in shorter operation times and less blood loss. No significant differences were found in mortality or complication rates. **Conclusions**: ORIF is associated with shorter operation times, less bleeding, and comparable in-hospital outcomes in treating Vancouver B2/3 fractures in higher-risk geriatric patients compared to revision arthroplasty. The retrospective design and small sample size in the ORIF group are limitations of the study. Further studies with functional evaluation are still required.

## 1. Background

Total hip arthroplasty (THA) represents a standard treatment for end-stage hip osteoarthritis and is considered one of the most successful orthopedic procedures. From 2006 to 2014, there was a 69.5% increase in primary THA and a 28.5% increase in revision THA in the United States [1]. Periprosthetic femoral fractures (PPFFs) are the third most common reason for revision after primary THA [2], with low-energy trauma, such as falls from standing height, accounting for about 85% of all cases [3]. The mean age of patients with PPFFs is approximately 80 years [4], with a mortality rate of 4.8% within 90 days and 13.4% within one year of the fracture [5]. PPFFs have a massive impact on daily life, with about 15% of patients being unable to return home after discharge [6].

The Vancouver classification system, introduced by Duncan and Masri [7], categorizes PPFFs into types A, B, and C. Type A fractures involve the trochanteric area and are subclassified into Ag (greater trochanter) and Al (lesser trochanter). Type B fractures occur around or just below the stem and are subclassified into B1 (stable stem), B2 (loose stem without substantial bone loss), and B3 (loose stem with substantial bone loss). Type C fractures are located below the stem with a stable prosthesis.

Although the management of PPFFs is primarily based on the Vancouver classification and fracture morphology, there is ongoing debate regarding the optimal surgical approach for treating Vancouver B2 and B3 fractures, which involve a loose femoral stem [8]. These fractures are particularly challenging due to the need to address both the fracture itself and the compromised stability of the prosthesis. Most experts advocate treating Vancouver B2 and B3 fractures with femoral component revision using a longer stem bypassing the fracture to achieve stability, which provides immediate postoperative stability, allowing for early weight-bearing, which is advantageous for patient mobility. However, it can lead to longer operative times, higher blood loss, and increased perioperative morbidity, particularly in high-risk patients [9,10,11,12,13]. However, some authors suggest that open reduction and internal fixation (ORIF) could be a viable alternative in certain situations, particularly for elderly patients with multiple comorbidities, as it may result in shorter operation times, fewer transfusions, and ultimately reduced overall complication rates [9,14,15,16,17]. On the other hand, delayed weight-bearing protocols can lead to prolonged rehabilitation and potential complications, such as non-union or implant failure [11,18].

This study aims to retrospectively compare the in-hospital postoperative outcomes in elderly patients with Vancouver B2 and B3 periprosthetic fractures who were treated with ORIF or femoral revision arthroplasty.

## 2. Methods and Materials

The study protocol was approved and registered by the local ethics committee (protocol No. 20-0247). This retrospective observational study was conducted at a level I trauma center in a major German city. We retrospectively enrolled patients treated operatively due to Vancouver B2 and B3 periprosthetic hip fractures from 1 January 2017 to 31 August 2023. Inclusion criteria featured adults over 65 years old with periprosthetic femur fractures after primary hip arthroplasty, which were operatively treated in our center. Exclusion criteria included patients under 65 years old, pathological fractures, perioperative PPFFs, re-revisions, and concurrent acetabular injuries like loosening of the acetabular component and periprosthetic acetabular fracture. The data with all relevant parameters and outcomes were well documented and complete from all patients. No patient was excluded, and there was no statistical procedure due to missing data.

Patient data were retrieved from the hospital’s database (Meona Ltd., Freiburg, Germany), anonymized, and analyzed in a confidential database (Microsoft Excel for Mac 2024, Version 16.89.1, Microsoft Corporation, WA, USA). Demographic parameters, such as age, gender, and body mass index (BMI) were recorded. In addition, preoperative comorbidities, such as hypertension, diabetes, osteoporosis, coronary artery disease, heart insufficiency, atrial fibrillation, chronic obstructive pulmonary disease, and dementia, as well as the status of the American Society of Anesthesiologists (ASA) were collected. Trauma mechanisms were drawn from the emergency report. Fall from standing height or less was considered a low-energy trauma. The collected surgical parameters included the type of treatment (revision of femoral components or ORIF), length of surgery, and units of red blood cell (RBC) transfusion. The revisions of femoral components were performed exclusively by using MUTARS^®^ (Implantcast, Buxtehude, Germany), MRP-TITAN^®^ (Peter Brehm, Weisendorf, Germany), and VerSys^®^ hip system (Zimmer Biomet, Warsaw, IN, USA). Osteosynthesis included the use of a plate osteosynthesis, cerclage, screw, or a combination of these three techniques. All cases of osteosynthesis received open reduction using a lateral or direct anterior approach. Minimally invasive percutaneous plating (MIPO) techniques were limited and occasionally performed only for the distal screw placement in order to reduce the incision length and were largely dependent on the fracture level. Bone grafting was used depending on the intraoperative grading of bony defects.

The Vancouver classification, as described previously, was used to categorize PPFFs, which was proved by a consultant radiologist. The decision to perform either revision arthroplasty or ORIF in patients with Vancouver B2 or B3 fracture was made by internal consensus of a team of surgeons from the field of arthroplasty and traumatology. Generally, revision arthroplasty is the gold standard to achieve stability. For patients who were treated by ORIF, it was determined that the fracture fragments were reducible, and the patient would not be able to tolerate a more extensive procedure like revision arthroplasty. The exception was that, by the chance of severe comminuted fracture or polished tapered-slip stem with unstable cement-stem-interface, revision arthroplasty was favored. All ORIF procedures were performed by a team of experienced trauma surgeons (consultant), while revision arthroplasty procedures were conducted by arthroplasty specialists (consultant). In some cases, the teams collaborated based on the complexity of the fracture.

The in-hospital postoperative outcomes within the acute management were collected and compared between the two techniques. The timepoint for non-intraoperative outcomes was set at the time of discharge from acute management. According to our result, the length of hospital stay was 15.8 ± 7.9 days. For this length of hospital stay, ICU necessity, intraoperative blood loss (ml), transfusion necessity during the hospital stay, immobility rate at discharge, mortality rate, and surgical and non-surgical complication rate were evaluated. ICU necessity was positive when a patient with Vancouver B2/3 fracture went to ICU either directly after the surgery or during the hospital stay. The length of hospital stay was the days that a patient spent from admission in the emergency room to discharge from our center to further ambulant treatment. The daily postoperative mobilization achievements were documented. Principally, weight-bearing as tolerant was given to both groups of patients from ORIF and arthroplasty. Limitation of flexion till 90° and avoidance of leg-crossing were necessary after arthroplasty to prevent dislocation. Redon-Drainages were placed in both groups of patients receiving ORIF and arthroplasty as routine. The removal of redon-drainage depended on the daily drainage volume. Immobility was confirmed when the patients could not walk with aid at discharge and was documented by our physiotherapist as lying, sitting, and standing.

The primary outcomes of this study were immobility rate and mortality rate in the in-hospital setting. The secondary outcomes were operation time, blood loss, transfusion necessity, length of hospital stay, ICU necessity, and complications.

Age-and-gender-matched analysis and subgroup analysis of ASA 3 and 4 patients were conducted using the MatchIt package from R version 4.0.5. SPSS version 29.0 (SPSS Inc., Chicago, IL, USA) for statistical analysis. Categorical data were compared using Fischer’s exact test or the Pearson chi-square test depending on the sample size and expected cell count, and they are presented as percentages. The Kolmogorov–Smirnov test was performed to verify the normality of parametric data, which was presented with an average ± standard deviation. If normality was confirmed, a 2–sided student *t*–test was used to determine the difference between the two groups; if not, the 2–sided Mann–Whitney U test was applied. The paired student *t*–test was performed for parametric data in the age-gender-matched analysis after proof of normality. A one-way analysis of variance with the Tukey test was carried out to compare the intergroup statistical significance among the three groups. Statistical significance was determined with a *p*-value of <0.05. The data set was complete so that no handling of missing data was performed.

## 3. Results

A total of 133 patients who suffered Vancouver B2/3 fractures were consecutively enrolled in the current study. The mean age was 80.3 ± 10.0 years, ranging from 65 to 100 years. No significant difference in age and BMI was observed between the groups (*p* = 0.82). Fragility fractures with low-energy trauma represented the most typical mechanism, accounting for 91.7% of the cases (n = 122). Notably, 15% of the population had dementia (n = 20). Table 1 provides an overview of the study population.

Table 2 summarizes the surgical-related parameters for acute management of Vancouver B2/3 fractures. Vancouver B2 and B3 fractures were primarily managed with femoral component revision (76.5% and 87.5%, respectively). The average operation time for Vancouver B3 fractures was significantly longer (221.9 ± 70.8 min) compared to B2 fractures (158.0 ± 58.8 min, *p* < 0.05). A higher proportion of patients with Vancouver B3 fractures (87.5%) underwent femoral component revision compared to those with B2 fractures (76.5%), though this difference did not reach statistical significance. Notably, more than one-quarter of the patients remained immobile after surgery, regardless of whether they underwent ORIF or revision arthroplasty.

Focusing on the treatment strategies, 107 patients with Vancouver B2/3 fractures underwent revision arthroplasty, while 26 were treated with ORIF (Table 3). An age- and gender-matched analysis of patients with Vancouver B2/3 fractures (Table 4) revealed that ORIF was more frequently performed in patients with higher ASA grades (3 and 4) compared to revision arthroplasty (revision arthroplasty: 61.5% vs. ORIF: 88.5%, *p* = 0.03). ORIF was also associated with significantly shorter operation times (revision arthroplasty: 181.2 ± 65.8 min vs. ORIF: 135.4 ± 78.6 min, *p* = 0.03). Additionally, patients undergoing ORIF had less blood loss (revision arthroplasty: 1100.4 ± 745.3 vs. ORIF: 724.6 ± 859.3, *p* = 0.01) and were less likely to require a blood transfusion (revision arthroplasty: 23 vs. ORIF: 16, *p* = 0.03). The overall complication rate was significantly higher in patients receiving arthroplasty than ORIF (Arthroplasty 80.8% vs. ORIF: 53.9%, *p* = 0.04). However, no statistically significant differences were found between the two treatment strategies in terms of ICU necessity, length of hospital stay, postoperative immobility, mortality, and revision rate.

Further, a subgroup analysis of ASA 3 and ASA 4 patients with Vancouver B2/3 fractures was conducted (Table 5). ORIF resulted in shorter surgery times (revision arthroplasty: 179.3 ± 58.1 min vs. ORIF: 135.1 ± 81.1 min, *p* < 0.01), less blood loss (revision arthroplasty: 1262.3 ± 674.8 vs. ORIF: 706.1 ± 887.5, *p* = 0.003), and reduced transfusion requirements (revision arthroplasty: 90.6% vs. ORIF: 65.2%, *p* < 0.01) compared to revision arthroplasty. Although the length of hospital stay was shorter in patients undergoing ORIF (14.9 ± 12.7 days vs. 18.6 ± 10.5 days), this difference did not reach statistical significance (*p* = 0.13). Mortality rates were comparable between the two groups, with a slightly better outcome for patients after revision arthroplasty (*p* = 0.12). However, patients treated by ORIF experienced less non-revision-related complications but without statistical significance (*p* = 0.19). There was also no significant difference observed in postoperative ICU necessity (*p* = 0.36) and postoperative immobility (*p* = 0.10) between the two treatment strategies.

The documented complications in treating Vancouver B2/3 fractures are listed and ranked by frequency in Table 6. Femoral component dislocations represented the most common cause of reoperation in the revision arthroplasty group. This was followed by iatrogenic femoral fractures, postoperative hematomas, wound-healing disorders, and early periprosthetic joint infections. In the ORIF group, complications included two cases of implant malpositioning and one case of postoperative compartment syndrome, all of which required surgical revision. Similar patterns of non-surgical complications were found in both groups. Among these, postoperative urinary tract infections were the most frequent complication in patients undergoing revision arthroplasty. Cardiac decompensation, pleural effusion, and acute kidney failure were observed in both groups. Notably, cardiac decompensation was the leading cause of death, with 13 cases documented across both treatment groups.

## 4. Discussion

The current study retrospectively evaluated the acute management of Vancouver type B2 and B3 fractures in a level I university trauma center, involving 133 recruited patients, including 85 and 48 cases of Vancouver B2 and B3 fractures, respectively. An essential finding of this study is that treating B2 or B3 fractures with ORIF was not associated with delayed mobilization and inferior mortality rate in an in-hospital setting. Moreover, it was associated with significantly shorter operation times and less intraoperative bleeding compared to femoral component revision. As the demand for THA continues to grow because of the higher life expectancy and increasing demand for functional mobility, more PPFFs and more geriatric patients are likely to be encountered in the near future [19]. Risk factors for periprosthetic fractures include advanced age, osteoporosis, and comorbidities such as diabetes or cardiovascular disease. These factors are particularly relevant in the elderly population, where low-energy trauma, such as falls, is the leading cause of fractures [18]. Certain stem types can also increase the chance of PPFFs, such as cemented polished taper slip stem, also known as “axe splitter”, which has a higher incidence of PPFFs as cemented composite beam stem [20].

This study’s data revealed that the mean age of patients suffering PPFFs was 80 years. Geriatric trauma patients represent a significant challenge for acute medical management due to their increased comorbidities, longer medication lists, and relatively fewer physiological reserves [21]. ORIF benefits high-risk geriatric patients due to reduced operative times and fewer transfusions. However, it might be associated with delayed weight-bearing, complicating postoperative recovery [16]. Revision arthroplasty provides greater postoperative stability, enabling early mobilization. Yet, it entails longer surgical times, higher blood loss, and potentially greater perioperative morbidity [22]. In other words, revision arthroplasty should be considered in patients with higher functional demands, where immediate weight-bearing is a priority [10,11,18,23]. In our center, 19.5% of patients with type B2 and B3 fractures were treated with ORIF.

The indications for ORIF and femoral stem revision are often decided by the fracture morphology and implanted stem type. However, the decisions might also be largely influenced by the general condition of the patient. In previous studies evaluating the outcome of ORIF vs. revision arthroplasty in PPFFs with comparable patient cohorts regarding age and gender, there was an apparent increase in the choice of ORIF in Vancouver B Fractures in recent years [24]. Notably, in the age-and-gender-matched analysis, a bigger proportion of patients with ASA 3 or ASA 4 were found in the ORIF group in our study. This underlines that ORIF was tendentially chosen for patients with higher perioperative risks, disregarding the fracture morphology and stem type. The analysis of all patient cohorts showed a significantly higher mortality rate in ORIF groups. This is because the patients receiving ORIF were already known to have higher perioperative risk. In contrast, revision arthroplasty was preferred in treating Vancouver B2/3 to achieve absolute stability, especially in patients with more function demand. However, this statistical significance no longer existed in the subgroup analysis when only ASA 3 and ASA 4 patients were included.

At the same time, no statistical differences were found regarding postoperative complications, revision rates, and mobility. Consequently, the in-hospital perioperative outcomes of patients treated with ORIF compared to femoral component revision were comparable. Revision arthroplasty aims to facilitate rapid postoperative remobilization, as the revision prosthesis generally provides greater stability compared to ORIF. This often allows full weight-bearing immediately after surgery. However, the current study did not demonstrate a superior mobilization rate at discharge for revision arthroplasty. This may be attributed to longer operative times and iatrogenic trauma. Moreover, a previous study from our group indicated that elderly patients often struggle to maintain partial weight-bearing, which diminishes the advantages of revision arthroplasty in this specific patient population [25]. This fact also supports our finding that the common goal in treating Vancouver B2/3 fractures of rapid remobilization was not diminished by the ORIF compared to revision arthroplasty.

Revision arthroplasty is generally a more complex procedure compared to ORIF. Preserving the femoral stem during osteosynthesis, along with requiring less extensive surgical exposure, leads to shorter operation times and reduced blood loss in ORIF procedures [12,14,26]. Smitham et al. reported convincing outcomes of ORIF in treating B2 fractures and highlighted the importance of anatomical reduction in such cases [16]. The rationale for using ORIF alone to treat PPFFs with unstable stems is based on the ability of the double-tapered stem to regain stability in the cement mantle after the reduction of a B2/3 fracture due to the rectangular cross-section, which allows for less subsidence within the cement mantle [27]. Controversial opinions were given in cases of B2/3 fracture with a polished tapered-slip stem with an impaired cement–bone interface [28]. Many surgeons prefer arthroplasty in this case to bring absolute stability. New data, however, favored ORIF over arthroplasty, which had a lower incidence of postoperative complications [29]. Similar to the result presented in the current study, no significant difference in revision rates and complication rates for both methods of treating type B fractures was reported by Lewis et al. [30]. A similar retrospective study with a smaller sample size from Pombo-Alonso et al. also emphasized the feasibility of ORIF treating patients with severe comorbidities [22]. A recent multicenter cohort study also demonstrated that ORIF is a reliable alternative for selected patients with limited functional demand and higher perioperative risk for revision arthroplasty [31].

Complications during the acute management of Vancouver B2/3 fractures were listed in the current study. The femoral component dislocation was the most frequent complication, leading to a reoperation after revision arthroplasty. In the documented non-surgical complications, common complication patterns in geriatric trauma patients, such as urinary tract infections, pleural effusion, and acute renal failure, were found. Cardiac decompensation was the cause of all deaths related to the treatment of B2/3 fractures. Previous literature showed that interdisciplinary orthogeriatric care can significantly improve postoperative mobility after hip fractures [32]. Therefore, geriatricians’ involvement should always be considered when dealing with PPFFs.

Several limitations must be acknowledged. First, this is a single-center retrospective cohort study with a relatively small sample size. The smaller sample size in the ORIF group may reduce the statistical power to detect significant differences in mortality and complications. As a retrospective study, this analysis may be subject to selection bias and lacks the control of a randomized trial. This limitation is mainly due to the low incidence of Vancouver B2/3 fractures. The retrospective design of this study represents a second limitation, as surgical strategies were not randomly controlled. It is important to note that the primary contraindications for ORIF in treating B2/3 fractures are fracture behavior and stem type, although the patient’s perioperative risks also influence decision-making. This underscores the challenge of conducting a randomized trial, as the Vancouver classification alone may not be a sufficient indicator for treatment selection. Nonetheless, efforts were made in this study to enhance the robustness of the findings through age- and gender-matched analysis and subgroup analysis, providing a more comprehensive interpretation of the data. No logistic regression was conducted to handle the impact of comorbidities on the outcomes due to the lack of events per predictor. Instead, subgroup analysis using ASA classification was performed to control the comparability of the two groups. Lastly, follow-up with function comparison was not included, as this study focused on the short-term outcomes of primary management, which is consistent with its specific hypothesis.

In conclusion, ORIF in the primary management of Vancouver B2/3 fractures is associated with shorter operation times, less blood loss, and comparable in-hospital postoperative outcomes compared to revision arthroplasty. Therefore, ORIF is a considerable alternative for the treatment of Vancouver B2/3 fractures, especially in geriatric patients with higher perioperative risk. However, further research with larger sample sizes and longer follow-up periods is required to confirm these findings.

## Figures and Tables

**Table 1:** Baseline clinic parameters.

	Total	Vancouver B2	Vancouver B3
n	133	85	48
	Mean ± SD	Mean ± SD	Mean ± SD
Age	80.3 ± 10.0	79.3 ± 9.7	82.6 ± 8.8
BMI	24.5 ± 4.8	25.1 ± 4.2	24.0 ± 5.1
	n (%)	n (%)	n (%)
Sex (female)	81 (60.9)	47 (55.3)	34 (70.8)
Low-energy trauma	122 (91.7)	76 (89.4)	46 (95.8)
ASA 1	3 (2.3)	2 (2.4)	1 (2.1)
ASA 2	32 (24.1)	23 (27.1)	9 (18.8)
ASA 3	84 (63.2)	52 (61.2)	32 (66.7)
ASA 4	15 (11.3)	8 (9.4)	7 (14.6)
Hypertension	84 (63.2)	51 (60.0)	33 (68.8)
Diabetes	15 (11.3)	10 (11.8)	5 (10.4)
Osteoporosis	32 (24.1)	20 (23.5)	12 (25.0)
CAD	22 (16.5)	10 (11.8)	12 (25.0)
Heart insufficiency	14 (10.5)	9 (10.6)	5 (10.4)
Atrial fibrillation	35 (26.3)	21 (24.7)	14 (29.2)
COPD	10 (7.5)	3 (3.5)	7 (14.6)
Dementia	20 (15.0)	14 (16.5)	6 (12.5)

ASA = American Society of Anesthesiologists; BMI = body mass index; CAD = coronary artery disease; COPD = chronic obstructive pulmonary disease; SD = standard deviation.

**Table 2:** Parameters of acute patient management.

	Total	Vancouver B2	Vancouver B3
n	133	85	48
	Mean ± SD	Mean ± SD	Mean ± SD
Operation time (min)	165.8 ± 70.0	158.0 ± 58.8	221.9 ± 70.8
Length of hospital stay (d)	15.8 ± 7.9	16.6 ± 10.1	16.6 ± 5.6
	n (%)	n (%)	n (%)
ORIF	26 (19.5)	20 (23.5)	6 (12.5)
Revision arthroplasty	107 (80.5)	65 (76.5)	42 (87.5)
Immobility	36 (27.1)	22 (25.9)	14 (29.1)
Mortality	11 (8.3)	8 (9.0)	3 (6.3)

ORIF = open reduction and internal fixation; SD = standard deviation.

**Table 3:** Comparison between femoral component revision and osteosyntheses in treatment of Vancouver B2/3 periprosthetic fracture.

Procedure	Revision Arthroplasty	ORIF	*p*-Value
n	107	26	
	Mean ± SD	Mean ± SD	
Age	80.4 ± 8.8	80.9 ± 12.0	0.40
Operation Time (min)	**192.2 ± 63.7**	**135.4 ± 78.6**	**<0.01**
Length of hospital stay (d)	17.1 ± 8.1	14.7 ± 10.9	0.10
Blood loss (mL)	**1283.2 ± 782.5**	**724.6 ± 859.3**	**0.003**
	n (%)	n (%)	
RBC transfusion necessity	**91 (85.0)**	**16 (61.5)**	**0.03**
ICU necessity	31 (29.0)	8 (30.8)	0.22
Immobility	28 (26.2)	8 (30.8)	0.36
Mortality	**6 (5.6)**	**5 (19.2)**	**0.02**
Complications	**82 (76.6)**	**14 (53.8)**	**0.02**
Non-revision-related complications	61 (57.0)	11 (42.3)	0.19
Revision	21 (19.6)	3 (11.5)	0.34

RBC = red blood cell; ICU = intensive care unit.

**Table 4:** Age-gender-matched comparison between femoral component revision and osteosyntheses in treatment of Vancouver B2/3 periprosthetic fracture.

Procedure	Revision Arthroplasty	ORIF	*p*-Value
n	26	26	
	Mean ± SD	Mean ± SD	
Age	80.9 ± 10.1	80.9 ± 12.0	1.00
Operation Time (min)	**181.2 ± 65.8**	**135.4 ± 78.6**	**0.03**
Length of hospital stay (d)	14.1 ± 4.6	14.7 ± 10.9	0.81
Blood loss (mL)	**1100.4 ± 745.3**	**724.6 ± 859.3**	**0.01**
	n (%)	n (%)	
Female	20 (76.9)	20 (76.9)	1.00
ASA 3 and ASA 4	**16 (61.5)**	**23 (88.5)**	**0.03**
RBC transfusion necessity	**23 (88.5)**	**16 (61.5)**	**0.03**
ICU necessity	6 (23.1)	8 (30.8)	0.53
Immobility	7 (26.9)	8 (30.8)	0.76
Mortality	1 (3.8)	5 (19.2)	0.19
Complications	**21 (80.8)**	**14 (53.9)**	**0.04**
Non-revision-related complications	15 (57.7)	11 (42.3)	0.27
Revision operation	6 (23.1)	3 (11.5)	0.47

ASA = American Society of Anesthesiologists; RBC = red blood cell; ICU = intensive care unit; SD = standard deviation.

**Table 5:** Subgroup analysis of ASA3 and ASA4 patients with Vancouver B2/3 fracture.

Procedure	Revision Arthroplasty	ORIF	*p*-Value
n	64	23	
	Mean ± SD	Mean ± SD	
Age	81.7 ± 8.7	81.4 ± 11.7	0.79
Operation Time (min)	**179.3± 58.1**	**135.1 ± 81.1**	**0.006**
Length of hospital stay (d)	18.2 ± 0.1	14.6 ± 11.3	0.13
Blood loss (mL)	**1262.3 ± 674.8**	**706.1 ± 887.5**	**0.003**
	n (%)	n (%)	
RBC transfusion necessity	**58 (90.6)**	**15 (65.2)**	**0.01**
ICU necessity	18 (28.1)	8 (34.8)	0.36
Immobility	20 (31.3)	8 (34.8)	0.10
Mortality	5 (7.8)	5 (21.7)	0.12
Complications	46 (71.9)	13 (56.5)	0.17
Non-revision-related complications	38 (59.4)	10 (43.5)	0.19
Revision	8 (12.5)	3 (13.0)	0.94

RBC = red blood cell; ICU = intensive care unit; SD = standard deviation.

**Table 6:** Ranking of documented complications after revision or ORIF in patients with Vancouver B2/B3 fractures.

	Revision Arthroplasty (n = 107)	Count	ORIF (n = 26)	Count
Revision-related complications	Femoral component dislocation	8	Implant malposition	2
	Iatrogenic femoral fracture	5	Compartment syndrome	1
	Postoperative hematoma	3		
	Wound-healing disorder	3		
	Early periprosthetic joint infection	2		
	Femoral component loosening	1		
Non-surgical complication	Urinary tract infection	16	Urinary tract infection	4
	Cardiac decompensation	9	Cardiac decompensation	4
	Pleural effusion	5	Pleural effusion	2
	Acute kidney failure	5	Acute kidney failure	2
	Pulmonary embolism	4		
	Wound-healing disorder	3		
	Pneumonia	3		
	Deep venous thrombosis	2		
	Respiratory failure	2		
	Ileus	1		

ORIF = open reduction and internal fixation.

## Data Availability

The data presented in this study are available on request from the corresponding author.

## References

[1] Patel I., Nham F., Zalikha A.K., El-Othmani M.M. (2023). Epidemiology of total hip arthroplasty: Demographics, comorbidities and outcomes. Arthroplasty.

[2] Gwam C.U., Mistry J.B., Mohamed N.S., Thomas M., Bigart K.C., Mont M.A., Delanois R.E. (2017). Current Epidemiology of Revision Total Hip Arthroplasty in the United States: National Inpatient Sample 2009 to 2013. J. Arthroplast..

[3] Lindahl H. (2007). Epidemiology of periprosthetic femur fracture around a total hip arthroplasty. Injury.

[4] (2022). The Compose Study Team. Epidemiology and characteristics of femoral periprosthetic fractures: Data from the characteristics, outcomes and management of periprosthetic fracture service evaluation (COMPOSE) cohort study. Bone Jt. J..

[5] Lamb J.N., Nix O., Al-Wizni A., West R., Pandit H. (2022). Mortality After Postoperative Periprosthetic Fracture of the Femur After Hip Arthroplasty in the Last Decade: Meta-Analysis of 35 Cohort Studies Including 4841 Patients. J. Arthroplast..

[6] Bottle A., Griffiths R., White S., Wynn-Jones H., Aylin P., Moppett I., Chowdhury E., Wilson H., Davies B.M. (2020). Periprosthetic fractures: The next fragility fracture epidemic? A national observational study. BMJ Open.

[7] Masri B.A., Meek R.M., Duncan C.P. (2004). Periprosthetic fractures evaluation and treatment. Clin. Orthop. Relat. Res..

[8] Patsiogiannis N., Kanakaris N.K., Giannoudis P.V. (2021). Periprosthetic hip fractures: An update into their management and clinical outcomes. EFORT Open Rev..

[9] Abdel M.P., Lewallen D.G., Berry D.J. (2014). Periprosthetic femur fractures treated with modular fluted, tapered stems. Clin. Orthop. Relat. Res..

[10] González-Martín D., Pais-Brito J.L., González-Casamayor S., Guerra-Ferraz A., Ojeda-Jiménez J., Herrera-Pérez M. (2022). Treatment algorithm in Vancouver B2 periprosthetic hip fractures: Osteosynthesis vs revision arthroplasty. EFORT Open Rev..

[11] González-Martín D., Hernández-Castillejo L.E., Herrera-Pérez M., Pais-Brito J.L., González-Casamayor S., Garrido-Miguel M. (2023). Osteosynthesis versus revision arthroplasty in Vancouver B2 periprosthetic hip fractures: A systematic review and meta-analysis. Eur. J. Trauma Emerg. Surg..

[12] Di Martino A., Brunello M., Villari E., D’agostino C., Cosentino M., Bordini B., Rivera F., Faldini C. (2024). Stem revision vs. internal fixation in Vancouver B2/B3 periprosthetic hip fractures: Systematic review and metanalysis. Arch. Orthop. Trauma Surg..

[13] Haider T., Hanna P., Mohamadi A., Merchan N., McNichol M., Wixted J.J., Appleton P.T., Nazarian A., von Keudell A.G., Rodriguez E.K. (2021). Revision Arthroplasty Versus Open Reduction and Internal Fixation of Vancouver Type-B2 and B3 Periprosthetic Femoral Fractures. JBJS Rev..

[14] Baum C., Leimbacher M., Kriechling P., Platz A., Cadosch D. (2019). Treatment of Periprosthetic Femoral Fractures Vancouver Type B2: Revision Arthroplasty Versus Open Reduction and Internal Fixation With Locking Compression Plate. Geriatr. Orthop. Surg. Rehabil..

[15] Jones A.R., Williams T., Paringe V., White S.P. (2016). The economic impact of surgically treated peri-prosthetic hip fractures on a university teaching hospital in Wales 7.5-year study. Injury.

[16] Smitham P.J., Carbone T.A., Bolam S.M., Kim Y.S., Callary S.A., Costi K., Howie D.W., Munro J.T., Solomon L.B. (2019). Vancouver B2 Peri-Prosthetic Fractures in Cemented Femoral Implants can be Treated with Open Reduction and Internal Fixation Alone without Revision. J. Arthroplast..

[17] Solomon L.B., Hussenbocus S.M., Carbone T.A., Callary S.A., Howie D.W. (2015). Is internal fixation alone advantageous in selected B2 periprosthetic fractures?. ANZ J. Surg..

[18] Perry M., Rivera J.-L., Wesolowski M., Eikani C., Cohen J., Brown N., Lack W.D. (2023). Treatment of Vancouver B2 Femur Fractures with Open Reduction Internal Fixation Versus Revision Arthroplasty. Cureus.

[19] Shichman I., Roof M., Askew N., Nherera L., Rozell J.C., Seyler T.M., Schwarzkopf R. (2023). Projections and Epidemiology of Primary Hip and Knee Arthroplasty in Medicare Patients to 2040–2060. JBJS Open Access.

[20] Mabrouk A., Feathers J.R., Mahmood A., West R., Pandit H., Lamb J.N. (2024). Systematic Review and Meta-Analysis of Studies Comparing the Rate of Post-operative Periprosthetic Fracture Following Hip Arthroplasty with a Polished Taper Slip versus Composite Beam Stem. J. Arthroplast..

[21] Southern A.P., Lopez R.A., Jwayyed S. (2024). Geriatric Trauma. StatPearls.

[22] Pombo-Alonso S., Gabarain I., Nunes N., De la Herran G. (2024). Managing B2 periprosthetic femoral fractures: ORIF vs stem-revision. Injury.

[23] Barghi A.M., Hanna P., Merchan N., Lechtig A., Haggerty C., Weaver M.J., von Keudell A., Wixted J., Appleton P., Rodriguez E. (2022). Outcomes After Operative Fixation of Vancouver B2 and B3 Type Periprosthetic Fractures. J. Orthop. Trauma.

[24] Laurer H.L., Wutzler S., Possner S., Geiger E.V., El Saman A., Marzi I., Frank J. (2011). Outcome after operative treatment of Vancouver type B1 and C periprosthetic femoral fractures: Open reduction and internal fixation versus revision arthroplasty. Arch. Orthop. Trauma Surg..

[25] Kammerlander C., Pfeufer D., Lisitano L.A., Mehaffey S., Böcker W., Neuerburg C. (2018). Inability of Older Adult Patients with Hip Fracture to Maintain Postoperative Weight-Bearing Restrictions. J. Bone Jt. Surg. Am..

[26] Joestl J., Hofbauer M., Lang N., Tiefenboeck T., Hajdu S. (2016). Locking compression plate versus revision-prosthesis for Vancouver type B2 periprosthetic femoral fractures after total hip arthroplasty. Injury.

[27] Scheerlinck T., Casteleyn P.P. (2006). The design features of cemented femoral hip implants. J. Bone Jt. Surg. Br..

[28] Scott CE H., Jain S., Moran M., Haddad F.S. (2023). Which Unified Classification System type B periprosthetic fractures around cemented polished tapered stems should not be fixed?. Bone Jt. J..

[29] Jain S., Farook M.Z., Aslam-Pervez N., Amer M., Martin D.H., Unnithan A., Middleton R., Dunlop D.G., Scott C.E.H., West R. (2023). A multicentre comparative analysis of fixation versus revision surgery for periprosthetic femoral fractures following total hip arthroplasty with a cemented polished taper-slip femoral component. Bone Jt. J..

[30] Lewis D.P., Tarrant S.M., Cornford L., Balogh Z.J. (2022). Management of Vancouver B2 Periprosthetic Femoral Fractures, Revision Total Hip Arthroplasty Versus Open Reduction and Internal Fixation: A Systematic Review and Meta-Analysis. J. Orthop. Trauma.

[31] Biberthaler P., Pflüger P., Wurm M., Hanschen M., Kirchhoff C., Aderinto J., Whitwell G., Giannoudis P.V., Kanakaris N. (2022). Clinical and Radiological Outcome of Vancouver B2 Fracture Treated with Open Reduction and Internal Fixation. A Multicenter Cohort Analysis. J. Orthop. Trauma.

[32] Gleich J., Fleischhacker E., Rascher K., Friess T., Kammerlander C., Böcker W., Bücking B., Liener U., Drey M., Höfer C. (2021). Increased Geriatric Treatment Frequency Improves Mobility and Secondary Fracture Prevention in Older Adult Hip Fracture Patients-An Observational Cohort Study of 23,828 Patients from the Registry for Geriatric Trauma (ATR-DGU). J. Clin. Med..

